# Income, Relative Deprivation and the Self-Rated Health of Older People in Urban and Rural China

**DOI:** 10.3389/fpubh.2021.658649

**Published:** 2021-07-06

**Authors:** Wenzhe Qin, Lingzhong Xu, Shoucai Wu, Hui Shao

**Affiliations:** ^1^Centre for Health Management and Policy Research, School of Public Health, Cheeloo College of Medicine, Shandong University, Jinan, China; ^2^National Health Commission (NHC) Key Lab of Health Economics and Policy Research (Shandong University), Jinan, China; ^3^Department of Geriatrics, Cheeloo College of Medicine, Qilu Hospital, Shandong University, Jinan, China; ^4^Department of Pharmaceutical Outcomes and Policy, College of Pharmacy, University of Florida, Gainesville, FL, United States

**Keywords:** income, relative deprivation, self-rated health, elderly, China

## Abstract

**Background:** Substantial evidence indicated that absolute income is directly associated with health. Few studies have, however, examined if relative income may be equally associated with health. This study aimed to investigate the association between absolute income/relative deprivation (RD) and self-rated health (SRH). We also investigated whether the urban-rural difference was existing in these associations.

**Methods:** Using cross-sectional data of 7,070 participants in the Shandong Family Health Service Survey of older people, this study applied binary logistic model and semi-parametric model to estimate the effect of absolute income and relative deprivation on SRH of older people. The Kakwani Index was used as a measure of relative deprivation at the individual level.

**Results:** Absolute income has a significant positive effect on the SRH among both urban and rural older people. When considered RD as a variable, both absolute income and RD have negative significant effects on SRH among all older people. In addition, the negative effect of RD on rural elderly is more pronounced than that of urban older populations. Semi-parametric regression results show that there was a complex non-linear relationship between income and SRH. Psychological distress substantially attenuated the association between relative deprivation and SRH.

**Conclusions:** Relative deprivation is negatively associated with self-rated health in both urban and rural older people after controlling the absolute income. RD may partly explain the association between income inequality and worse health status. Compared with the urban elderly, the effect of income-based relative deprivation on SRH was more pronounced among the rural elderly, and more care should be given to the lower income and rural older populations.

## Introduction

The positive association of socioeconomic status with health has been well-established in the previous literature (1–3). As a main indicator of socioeconomic status, income has a strong impact on health. To a certain degree, the higher income people earn, the better their health (4). Two alternative hypotheses have been offered to explain the impact of income on individual's health. The absolute income hypothesis (AIH) suggested that absolute income has a direct effect on health (5, 6). Individuals with higher income are more likely to have good health than lower income individuals, because they have enough material resources that are conducive to good health. However, an individual' s health is not only determined by his own income, but also by relative income of others (7–9). Based on social comparison, the effect of relative income on health is referred to as the relative income hypothesis (RIH).

The RIH has several forms, such as income inequality, income rank and relative deprivation (RD) (10, 11). The impact of relative deprivation is one form of the RIH. Relative deprivation has been defined as “the difference between an individual's income and the income of individuals in their reference group” (12). Individuals tend to compare themselves with people whose income is higher than their own in the reference group. The greater the income gap, the stronger the perceived relative deprivation (13). Another form of the RIH is the income inequality which focuses on overall income deprivation. This measure is not considered in this study, although the findings are important for enriching our paper. There are two alternative pathways through which RD may affect one's health. One is material pathway, which suggested that RD increased the inequality of one's access to goods, services, and social activities such as employment or social networks and thereby adversely affects one's health (14). The other one is psychosocial pathway, which implies that those who feel relatively deprived will have some negative emotions, such as frustration or stress. The psychological stress may lead to directly negative effects on mental health or indirectly effects on physical well-being via health behaviors (e.g., smoking, alcoholism and substance use) (15, 16).

Previous studies which empirically tested the relative deprivation hypothesis have been presented the negative impacts of relative deprivation on various health outcomes. These negative effects are manifested in the increased prevalence of chronic diseases (17, 18), stress-related health behavior, such as smoking and drug abuse (19, 20) and poor self-rated health (21), functional disability (22) and mortality (23). However, the research results diverged, when considering different health outcomes. Studies utilizing self-rated health (SRH) as the outcome measure provided supportive evidence that RD has a strong association with SRH (24), findings from several studies focusing on other health outcomes (i.e., mortality and depression) did not (25, 26). In any case, almost all of these studies were conducted on developed countries, and few RIH related studies are conducted in low-income or middle-income countries.

China has witnessed rapid economic growth in the past 30 years, the per capita income of urban and rural residents in China has increased significantly. However, at the same time rapid economic growth has been accompanied by obvious income gap (27). Increasing income gap has brought negative impacts on the rapid increase in income inequality and individual quality of life, which ends to make vulnerable groups more vulnerable to adverse effects (28). Research and statistical data have shown that health and income levels have not increased simultaneously (29, 30). The unique dual structure of urban and rural areas makes income inequality present obvious regional differences (31). In contrast to the majority of the existing literature, which has been conducted in developed countries (15, 21, 24), the focus of this study is on a sample of older adults in China. This study focused on older people in urban and rural China for some reasons: First, China is rapidly transforming into an aging nation, and much attention has been paid to the health status of older adults (32). Secondly, it was hypothesized that relative deprivation based on income would be more pronounced in older populations. The incidence of poverty in this group is much higher than that of the general population (33). From a life course perspective, the impact of relative deprivation on health should be evidenced in older people because the influence of poverty on health may accumulate over time (34). In addition, there are obvious urban-rural differences in the living conditions and healthcare services of the older populations in China, and the relative deprivation is more prominent among them (35). Overall, this current study investigated the association between absolute income/relative deprivation and SRH. We also investigated whether the urban-rural difference was existing in the association between absolute income/relative deprivation and SRH.

## Methods

### Study Population

Data were collected from the 2017 Survey of the Elderly Family Health Service. The survey was conducted in Shandong province, China. Stratified multi-stage random sampling was applied: in the first stage, according to the level of socioeconomic development (high, medium, and low) and geographical location (east, central and west), using probability proportionate to size sampling method (PPS), 3 cities were selected from 17 cities as the primary sampling units (PSUs). From each PSU, 1 district and 1 county were selected as the secondary sampling units (SSUs), and represented urban and rural areas separately (PPS). In the third stage, three towns and three sub-districts were selected randomly from each county and district separately (PPS). Then, from each town and sub-district, six villages and six committees were selected separately (PPS). Lastly, an average of 50 households were randomly selected and making up the total sample (Simple random sampling). Eligible participants were those aged 60 years or older with local household registrations. Finally, a total of 5,643 households consisting of 7,070 individuals were included in the sample. All data collection was performed by trained master students in the participant's home using a self-administered questionnaire (SAQ). The Myer's Index was estimated to be 2.19, and test of goodness for fit was not statistically significant, indicating a good quality of sampled data.

#### Self-Rated Health

SRH is an effective and reliable measure of health (36). In our survey, self-rated health was assessed using a single item: “Generally, how would you rate your current health status?” It has a 5-point Likert scale (very good, good, fair, poor, very poor). Scores were reverse coded and treated as a continuous measure ranging from 1 (very poor) to 5 (very good). There were relatively few respondents who provided extreme responses (“very good” or “very poor”) to the health status question, in our analysis, SRH was dichotomized into two categories: 1 = good health, where SRH was either good or very good; 0 = poor health, where SRH was fair, poor or very poor. We modeled the probability of reporting good/very good (hereafter, good) health. Previous studies also divided SRH into two other categories: 1 = good health (very good/good/ fair); 0 = poor health (poor/very poor), and we carried out regression analysis according to this classification (see Tables C1, C2 of Supplementary Material).

#### Individual Income

Data on total individual income in the last 12 months was collected as continuous variable, which included farming income, fishing income, livestock income, retirement wages, pension, business income, children's support, investment income and other types of subsidies income. We make the assumption that an individual compares him/herself to others in his/her reference group based on their own income. When it comes to social comparisons, individuals are more likely to evaluate themselves in terms of their paychecks and other income and less likely to account for their household structure and the within household distribution of the total household income. Given that a considerable proportion of the participants are living in the same household, when it comes to possible common income (such as farming, fishing and livestock income. etc.), the individual income is determined by dividing the common income by the number of people surveyed in the family. The income structure for rural vs. urban participants was shown in Table A1 of Supplementary Material.

To ensure more accurate estimation of the non-linear relationship between absolute income and health, income was then transformed using logarithmic function, as suggested by previous work (37). Furthermore, logarithm of income also prevent bias on the coefficient on the relative income measures. Based on previous studies, the first hypothesis of our study is that actual income would be positively correlated with SRH.

#### Relative Deprivation

The Kakwani Index was used to measure RD. The Kakwani index is obtained on the basis of Yitzhaki index, which has the properties of dimensionless, normality and transfer invariance (38). Before measuring individual RD, it is necessary to give a reference group for individual comparison. Considering the urban-rural dual structure of China, we divided the total sample into rural and urban subgroups, and assume that individuals in each subgroup compare themselves with other individuals with higher income in the same group.

Formally, the Kakwani relative deprivation (KRD) index is defined as a function of the Yitzhaki index divided by the mean income of total sample in the reference group. The formula developed by Kakwani for measuring individual RD is:

$$KRD\left(x,x_{i}\right)=\frac{1}{n\mu_{X}}\sum_{j=i+1}^{n}\left(x_{j}-x_{i}\right)=\gamma_{x_{i}}^{+}\left[\frac{\left(\mu_{x_{i}}^{+}-x_{i}\right)}{\mu_{X}}\right]$$

where *X* represents a reference group and *n* was the total sample size in this group. *KRD*(*x, x*_*i*_) was the relative deprivation index for individual *i*, and *x*_*i*_ represents the income of individual i and *x*_*j*_ is the incomes of all individuals *j* whose incomes are higher than individual *i*'s; *μ*_*X*_ is the average income of all samples in the reference group. $\mu_{x_{i}}^{+}$ is the average income of the sample whose income was higher than *x*_*i*_ in the reference group. $\gamma_{x_{i}}^{+}$ is the percentage of samples whose income was higher than *x*_*i*_ in the reference group. The greater the gap between *x*_*i*_ and *x*_*j*_, individual *i* was hypothesized to feel more deprived. The second hypothesis of our study is that KRD is negatively correlated with SRH. The greater the degree of RD for an individual, the poorer their SRH status would be.

#### Covariates

According to previous empirical studies in China and other counties (1, 39, 40), we controlled age, gender, educational level, marital status, chronic disease, activities of daily living (ADL), psychological distress and personality trait at the individual level. Age was measured in chronological years; Education attainment was measured with the number of years spent in full time education. We divided education into three levels: no school (0 years), primary school (1–6 years), and junior school and above (at least 7 years); Marital status was categorized into married and others; chronic disease were dichotomized (yes or not) and self-reported, assessed by asking whether the participant was diagnosed with the following conditions: hypertension, diabetes, heart disease, stroke, COPD, cancer, and other. For measuring ADL of elderly, the Lawton and Brody Instrumental Activities of Daily Living Scale was used (41). The scale consists of 14 questions for evaluating self-maintenance, transportation utilization, medication behavior, housework activities and financial management among the elderly. Each question used 4-grade score with a range from 14 to 56, where higher scores indicate lower level of competence. The psychological was measured by The Kessler Psychological Distress Scale (K10 scale) (42). The scale consists of 10 questions and each question used a five-value response that was scored from five (all the time) through to one (none of the time). The maximum score is therefore 50, indicating severe distress, and the minimum score is 10, indicating no distress. Personality trait was measured using a single question: “how would you rate your personality traits?”. Three options were provided: extraversion, introversion and in between.

### Statistical Analysis

All analyses were stratified by urban-rural populations. We first present socio-demographic characteristics and self-rated health of our sample, and tested the statistical differences using the Chi square test for categorical variables and the Kruskal-Wallis test for abnormal distributed continuous variables. Second, the binary logistic model and semiparametric regression model were applied to estimate the associations between absolute income and relative deprivation and SRH of older people, and the urban-rural difference in the coefficients was compared. Third, the sensitive analysis using different reference group was conducted to test the robustness of the estimates. All the statistical analyses were performed using Stata version 15.0. *P*-values were 2-sided, and statistical significance was set at *P* = 0.05.

## Results

### Descriptive Analysis by Urban and Rural Populations

Table 1 shows the summary statistics of all participants stratified by prefecture of residence. Over 70% of the sample was in the young-old age group (60–74 years old). More than half was female. The majority of the individuals were married. Overall, the educational attainment was higher among urban respondents than rural ones.

**Table 1:** General characteristics of the older people according to residence.

**Variables**	**Urban (*****n*** **=** **2,080)**		**Rural (*****n*** **=** **4,990)**	
	***n***	**%**	***n***	**%**
Log income (mean, sd)	4.18	0.51	3.60	0.38
KRD (mean, sd)	0.39	0.27	0.54	0.21
K10 (mean, sd)	14.49	5.88	15.61	6.87
**Self-rate health**				
Fair/Poor/very poor	859	41.3	2,439	48.9
Good/very good	1,221	58.7	2,551	51.1
**Age group**				
60–74	1,609	77.4	3,883	77.8
75+	471	22.6	1,107	22.2
**Gender**				
Male	695	33.4	1,701	34.1
Female	1,385	66.6	3,289	65.9
**Educational attainment**
No school	240	11.5	1,947	39
Primary school	905	43.5	2,069	41.5
Junior school and above	935	45	974	19.5
**Marital status**				
Married	1,734	83.4	4,040	81
Others	346	16.6	950	19
**Chronic disease**				
Yes	1,414	68	3,625	72.6
No	666	32	1,365	27.4
**ADL, score**				
14	1,786	85.9	3,681	73.8
15–21	246	11.8	1,037	20.7
≥22	48	2.3	272	5.5
**Personality trait**				
Extraversion	1,047	50.3	2,463	49.4
Introversion	570	27.4	1,548	31.0
In between	463	22.3	979	19.6

*KRD, Kakwani relative deprivation; ADL, activities of daily living; K10, The Kessler Psychological Distress Scale*.

Compared to urban participants, rural older people were more likely to have chronic disease and poorer ADL. The urban residents were more likely to report good/very good health compare with their rural counterparts (58.7 vs. 51.1%). Urban respondents had higher incomes than rural respondents, while the relative deprivation were more pronounced in rural elderly. The results indicated that the income gap was large within the rural area.

### Association of Absolute Income and Relative Deprivation With SRH

The absolute income and RD were highly correlated in rural and urban populations (*r* = −0.862, *P* < 0.001; *r* = −0.942, *P* < 0.001, respectively), which indicated that the lower the absolute income, the more severe the relative deprivation of the older people (especially among urban population). There is no multicollinearity among covariates in these two samples (see Table B1 of Supplementary Material). The results of logistic regression and semiparametric regression after controlling for covariates were shown in Tables 2, 3. We only reported a sub-set of the results, which focused on the impacts of absolute income and relative deprivation on SRH. The complete regression results are reported in the Tables B2, B3 of Supplementary Material. Before KRD index was included in the model, the regression coefficients of absolute income were positive and statistically significant for both urban and rural populations (Model 1 and Model 5). However, the association between absolute income and good SRH has reversed after including KRD index into the model, and the coefficients were statistically significant and negative (Model 2 and Model6). At the same time, there is also a significant negative correlation between KRD index and good SRH in both rural and urban samples (Model 2, Model 4, Model 6 and Model 8). This indicates that the huge negative association between RD and health pulls the coefficient of absolute income from positive to negative.

**Table 2:** Relationship between relative deprivation and SRH in urban older populations (select results)^a^^,^^b^^,^^c^.

**Variables**	**Model 1**	**Model 2**	**Model 3**	**Model 4**
Log income	0.273^*^ (0.105)	−0.143^*^ (0.295)	−0.080^*^ (0.302)	
KRD		−0.435^**^ (0.164)	−0.339^*^ (0.167)	−0.294^*^ (0.161)
K10, score			−0.078^***^ (0.009)	−0.017^***^ (0.002)

*KRD, Kakwani Relative Deprivation; K10, The Kessler Psychological Distress Scale*.

a *The coefficients in Model 1, 2, 3 were estimated by Binary logistic regression model and the coefficients in Model 4 was estimated by semiparametric regression model. Standard errors are in parentheses*.

b *The reference group was all the urban participants*.

c *All models were adjusted for age, gender, marital status, education chronic disease, ADL score and personality trait*.

* *P < 0.05*;

** *P < 0.01*;

*** *P < 0.001*.

**Table 3:** Relationship between relative deprivation and SRH in rural older populations (select results)^a^^,^^b^^,^^c^.

**Variables**	**Model 5**	**Model 6**	**Model 7**	**Model 8**
Log income	0.203^*^ (0.083)	−1.183^***^ (0.185)	−1.131^***^ (0.188)	
KRD		−3.026^***^ (0.347)	−2.736^***^ (0.353)	−0.504^***^ (0.062)
K10, score			−0.073^***^ (0.005)	−0.013^***^ (0.001)

*KRD, Kakwani Relative Deprivation; K10, The Kessler Psychological Distress Scale*.

a *The coefficients in Model 5, 6, 7 were estimated by Binary logistic regression model and the coefficients in Model 8 was estimated by semiparametric regression model. Standard errors are in parentheses*.

b *The reference group was all the rural participants*.

c *All models were adjusted for age, gender, marital status, education chronic disease, ADL score and personality trait*.

* *P < 0.05*;

^**^P < 0.01;

*** *P < 0.001*.

When comparing urban and rural populations, the absolute value of the regression coefficient of the KRD index on SRH among the rural older population was greater than that for the urban elderly, no matter in the logistic model or in the semiparametric model (−3.026 vs. −0.435; −0.504 vs. −0.294, respectively). This indicated that the impact of relative deprivation on SRH of rural elderly was more apparent than that of urban ones. To further test the psychosocial pathway that relative deprivation affecting SRH, we examined the changes of the regression coefficients of KRD index while controlling K10 scores in the models (Model 3 and Model 7). The findings showed that the coefficients of KRD index were attenuated in both urban and rural elderly, which indicated that psychological condition may play a mediating role in the relationship between relative deprivation and SRH. The models using alternative SRH categorization also showed similar results (see Tables C1, C2 of Supplementary Material).

**Figure 1:**
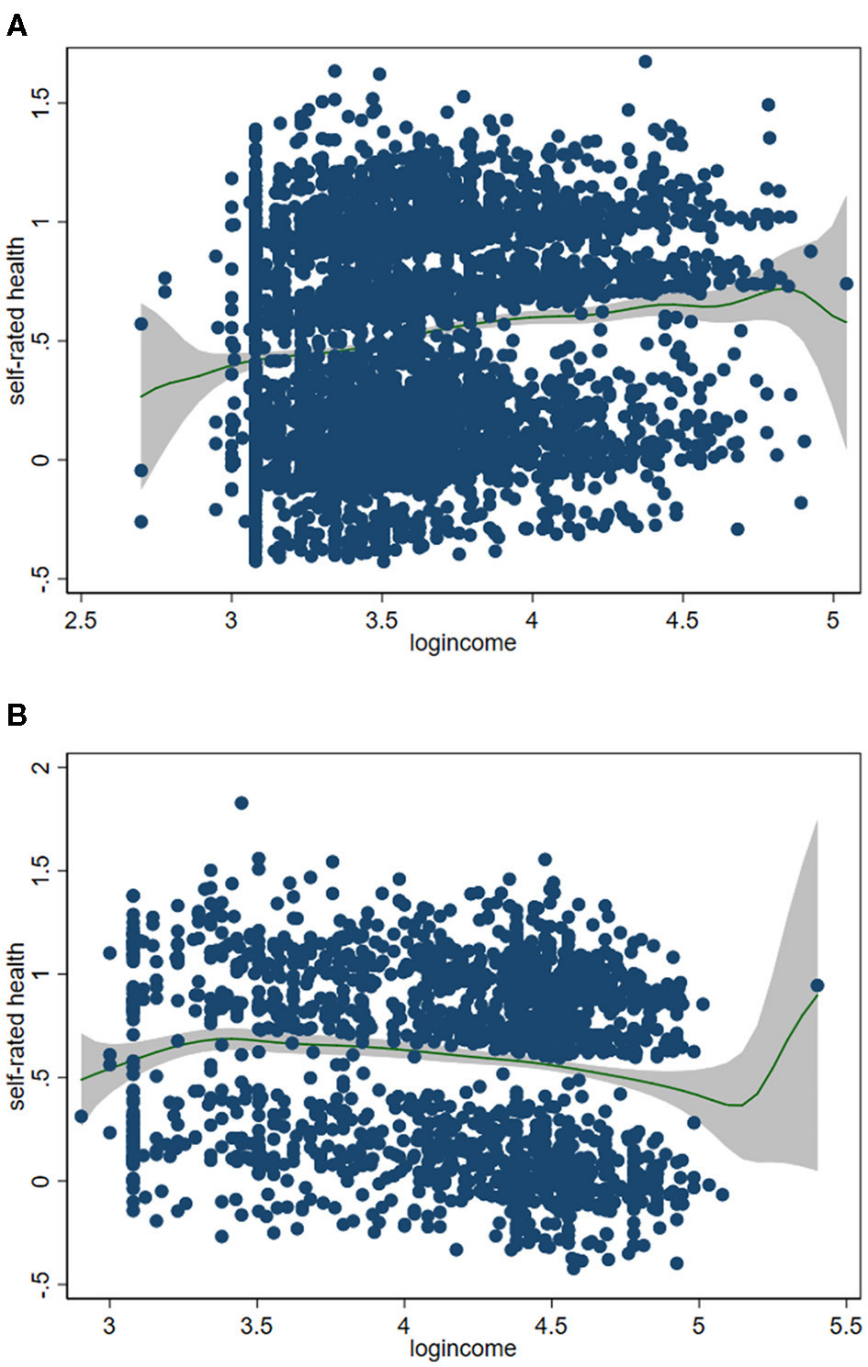
Relationship between absolute income and self-rated health in urban older populations **(A)** and rural older populations **(B)**^a, b^. ^a^Adjusted for age, gender, marital status, education, chronic disease, activities of daily living, personality trait, psychological distress and relative deprivation index. ^b^The absolute income was transformed using logarithmic function.

In order to further explore the relationship between income and SRH, we obtained kernel regression figures of the relationship between absolute income and SRH under the semiparametric model (Figure 1). The two figures showed that there was a complex non-linear relationship between income and SRH. The link between health and income at different levels of income is not straightforward. At low income levels, the absolute income has a positive association with the SRH for both rural and urban elderly, and the increase in income brought a steady improvement in health. However, at high income level, the impact of income on the SRH of both urban and rural elderly has declined and fluctuated. Meanwhile, the urban-rural differences appeared. In rural elderly, the SRH level decreased slightly when the logarithmic income was about 4.8, while in urban elderly, the SRH level decreased significantly when the logarithmic income was about 5.2, and then increased rapidly.

In addition, to investigated the association between RD and SRH when the reference group was a more granular units, we conducted a sensitivity analysis after replacing the reference group from the all rural participants (all urban participants) to the town (sub-district) where the participants were located. The sub-sets of the results of sensitivity analysis were shown in Tables 4, 5. The complete regression results are reported in the Online Appendix (see Tables B4, B5 of Supplementary Material). The results showed that there is still a negative correlation between KRD index and SRH in both rural and urban samples. The absolute value of the regression coefficient of the KRD index on SRH among the rural older population was still greater than that for the urban elderly, no matter in the logistic model or in the semiparametric model (−1.237 vs. −0.952; −0.252 vs. −0.179, respectively). As K10 scores was included in the model, the coefficients of KRD also decreased in both urban and rural elderly. The results of sensitivity analysis indicated that the association between RD and SRH was robust.

**Table 4:** Sensitive analysis in urban older population (select results)^a^^,^^b^^,^^c^.

**Variables**	**Model 2**	**Model 3**	**Model 4**
Log income	−0.336 (0.235)	−0.276^*^ (0.239)	
KRD	−0.952^*^ (0.396)	−0.874^*^ (0.403)	−0.179^**^ (0.161)
K10, score		−0.078^***^ (0.009)	−0.017^***^ (0.002)

*KRD, Kakwani Relative Deprivation; K10, The Kessler Psychological Distress Scale*.

a *The coefficients in Model 2, 3 were estimated by Binary logistic regression model and the coefficients in Model 4 was estimated by semiparametric regression model. Standard errors are in parentheses*.

b *The reference group was the urban older populations in the town/sub-district where the participant are located*.

c *All models were adjusted for age, gender, marital status, education chronic disease, ADL score and personality trait*.

* *P < 0.05*;

** *P < 0.01*;

*** *P < 0.001*.

**Table 5:** Sensitive analysis in rural older population (select results)^a^^,^^b^^,^^c^.

**Variables**	**Model 6**	**Model 7**	**Model 8**
Log income	−0.343^**^ (0.133)	−0.315^*^ (0.136)	
KRD	−1.237^***^ (0.232)	−0.992^**^ (0.238)	−0.252^***^ (0.046)
K10, score		−0.074^***^ (0.005)	−0.014^***^ (0.001)

*KRD, Kakwani Relative Deprivation; K10, The Kessler Psychological Distress Scale*.

a *The coefficients in Model 6, 7 were estimated by Binary logistic regression model and the coefficients in Model 8 was estimated by semiparametric regression model. Standard errors are in parentheses*.

b *The reference group was the rural older populations in the town/sub-district where the participant are located*.

c *All models were adjusted for age, gender, marital status, education chronic disease, ADL score and personality trait*.

* *P < 0.05*;

** *P < 0.01*;

*** *P < 0.001*.

## Discussion

Utilizing a new relative deprivation measure, the Kakwani Index, this study explored the impacts of absolute and relative income on self-rated health among Chinese older people. Four main findings were obtained: First, absolute income has a positive association with good SRH when relative income is not considered. Our study offered evidence to confirmed the AIH for both rural and urban older adults in China. Second, relative deprivation is associated with poor self-rated health. This result also demonstrates a support for the RIH in rural and urban Chinese elderly. Third, the magnitude of the correlation between relative income and SRH differed in rural and urban old populations. The impact of relative income on SRH was greater among the rural elderly compared to urban ones. Fourth, the present study shows that psychological distress substantially buffered the stronger negative impact of relative deprivation and SRH, suggesting that psychological condition may play a mediating role in the relationship between relative deprivation and SRH.

Empirical studies on the impact of absolute income on health have yielded mixed findings and there has been no consistent conclusion (43). However, the non-linear relationship between health and absolute income has often been reported (44, 45), and our results also offered evidence to confirm it. The result in our study that absolute income increased SRH at a decreasing rate was similar with previous research conducted in other countries and China (46, 47). People with higher household income, especially the older people, can increase access to healthcare by improving financial capacity to cover health-related expenses (48). In China, it seems that being poor can greatly reduce an individual's resilience to health shocks and lead to worse health (49). In addition, adequate finance will reduce future health vulnerability for the individual and the household by developing a balanced lifestyle, maintaining healthy living conditions and providing adequate resources for household dependents (50, 51). We only provided some possible explanations for the positive impact of absolute income on SRH, but considering the non-linear relationship between health and absolute income, the influential mechanism linking absolute income and health needs to be explored in future research.

This study also provided evidence to support the RIH in rural and urban Chinese older populations. The result reported a negative association between relative deprivation and SRH was in line with previous studies in China among adults and older populations (52, 53). An explanation of why relative deprivation in income may lead to poorer health status is the concept of allostatic load (54, 55). Invidious upward social comparisons often lead to perceived relative deprivation, and those who feel relatively deprived will have some negative emotions such as frustration and shame, thereby having a detrimental effect on mental health through the excessive secretion of the stress hormone, which leads to harmful health behaviors (56). Another explanation was the material pathway. Relative deprivation reduces the probability of individuals equally obtaining public goods, healthcare services and participating in social activities, thereby affecting the health of individuals (9).

The RIH in rural elderly was more pronounced than that in urban area. This urban-rural difference may be due to two reasons. First, the urban and rural elderly have different psychological perceptions of relative deprivation caused by income inequality. In China, income inequality in rural areas is higher than in urban areas, and income inequality aggravates the relative deprivation of rural populations (57). What's more, rural populations tend to be had lower socioeconomic status, which may lead to a strong sense of income inequality caused by income gap due to their living culture, and the idea of “suffering from poverty but not suffering from inequality” is more serious than urban residents. Second, differences in macro-structural characteristics of urban-rural segmentation makes the urban and rural elderly different in their tolerance for relative deprivation (58). Urban residents have relatively complete medical security, even if they are subject to greater income deprivation, they will suffer less psychological pressure. On the contrary, rural areas often lack medical resources, and the income deprivation suffered by rural residents will have a greater negative impact on their health. In addition, our results showed that the lower the absolute income, the more severe the relative deprivation of the older people, and low-income people in urban are more likely to feel relative deprivation. The lower the income of the elderly, the more difficult it is to meet their own needs, and they are more likely to be in a disadvantaged position when making social comparisons with others, resulting in relative deprivation. What's more, urban older people differ greatly in occupational levels, and there are obviously more high-educated and high-income groups than in rural areas. Therefore, when making social comparisons, urban low-income older people are more likely to have relative deprivation.

This study has a number of limitations. First, we lacked information on the actual reference groups people use to make social comparisons. Alternative reference groups based on age or education may have formed the basis for interpersonal comparisons. However, as a special group, the elderly gradually withdraws from the labor market, and the family income will reach the maximum at this time. The impact of age and education on income is no longer significant. Therefore, we assume that individuals in each subgroup (urban or rural) compare themselves with other individuals who have higher income in the same group. Second, we may have omitted some potential variables, such as individual variations in ability, temperament, and personality, which could also reflect the association between relative deprivation and health. We used an extensive range of control variables based on those used in previous literature, thereby reducing the chance of possible omitted variable bias. Third, the KRD Index is an objective measure of relative deprivation. It is not known whether those who had a higher KRD Index in fact perceived themselves as deprived compared to others. Previous studies have found that subjective feelings of deprivation or self-reported inequality was more important than objective measures (16). Fourth, a considerable proportion of the participants are living in the same household, and these people likely had a correlation in terms of SRH and income. Fifth, the data in our study is cross-sectional data, which can only reflect the current associations between income and relative deprivation and health, while delayed and cumulative effects cannot be reflected. Meanwhile, we are unable to demonstrate a causal relationship between relative deprivation and self-rated health because of the cross-sectional design.

## Conclusion

In conclusion, the present study supported the AIH and BIH in Chinese older adults. Relative deprivation is negatively associated with self-rated health in both urban and rural older people after controlling the absolute income. Furthermore, the correlation between relative income and SRH was more pronounced among the rural older populations than among the urban ones. Our findings have some potentially important policy implications. The first policy option is to improve the financial security by increasing income of older people, especially for the rural elderly. Second, the negative impact of relative deprivation could be addressed by reduce income inequality, such as via income transfers to reduce the gap between rich and poor. Third, when considering the impact of income on health, in addition to considering absolute income, the income-based relative deprivation should also be concerned.

## Data Availability Statement

The raw data supporting the conclusions of this article will be made available by the authors, without undue reservation.

## Ethics Statement

Written informed consent was obtained from the individual(s) for the publication of any potentially identifiable images or data included in this article.

## Author Contributions

LX and SW contributed to the conception and design of the study. WQ performed the statistical analysis and wrote the first draft of the manuscript. HS contributed to revise the paper. All authors contributed to the study design, critically reviewed draft versions and provided important intellectual content during revisions, and accept accountability for the overall work.

## Conflict of Interest

The authors declare that the research was conducted in the absence of any commercial or financial relationships that could be construed as a potential conflict of interest.
