# Supplementary material for: Income, Relative Deprivation and the Self-Rated Health of Older People in Urban and Rural China
Source: Front Public Health. 2021 Jul 6;9:658649. doi: 10.3389/fpubh.2021.658649 (PMC8291363; doi:10.3389/fpubh.2021.658649)
Supplement: Supplementary file 1 [file Table_1.DOCX]

Additional file 1: The distribution of Income resources for urban and rural participants.

Table A1 The distribution of income resources for urban and rural participants

| Income resources | Urban(n=2080) |  | Rural(n=4990) |
| --- | --- | --- | --- |
|  | (%) |  | (%) |
| Farming/fishing/livestock income | 0.32 |  | 16.28 |
| Wage income | 1.88 |  | 8.96 |
| Pension | 1.66 |  | 16.98 |
| Retirement wages | 83.87 |  | 28.18 |
| Children’s support | 6.71 |  | 18.35 |
| Investment income | 2.30 |  | 2.97 |
| Government relief fund | 0.18 |  | 1.23 |
| Business income | 0.17 |  | 2.56 |
| Others | 2.91 |  | 4.49 |

The income resource of the urban older populations is mainly from retirement wages, accounting for 83.87%, while the income resources of rural elderly are mostly from retirement wages (28.18%), children’s support (18.35%), pension subsidies (16.98%) and farming/fishing/livestock income (16.28%).

Additional file 2: Regression results of all variables from logistic models and semiparametric model(1=very good/good; 0=fair/poor/very poor)

Table B1 Multicollinearity test among covariates

| **variables** | Urban (n=2080) | |  | Rural (n=4990) | |
| --- | --- | --- | --- | --- | --- |
|  | Tolerance | VIF |  | Tolerance | VIF |
| Log income | 0.175 | 5.721 |  | 0.249 | 4.015 |
| KRD | 0.178 | 5.608 |  | 0.237 | 4.213 |
| K10 | 0.925 | 1.081 |  | 0.879 | 1.137 |
| Age group | 0.872 | 1.147 |  | 0.869 | 1.151 |
| Gender | 0.865 | 1.156 |  | 0.824 | 1.213 |
| Educational attainment | 0.797 | 1.255 |  | 0.795 | 1.258 |
| Marital status | 0.894 | 1.119 |  | 0.914 | 1.094 |
| ADL | 0.884 | 1.131 |  | 0.868 | 1.152 |
| Chronic disease | 0.963 | 1.039 |  | 0.938 | 1.066 |
| Personality trait | 0.987 | 1.013 |  | 0.983 | 1.017 |

Table B2 Association of absolute income and relative deprivation with SRH in urban older populations

| **Variable** |  | **Model 1** | **Model 2** | **Model 3** | **Model 4** |
| --- | --- | --- | --- | --- | --- |
| **Log income** |  | 0.273*(0.105) | -0.143*(0.295) | -0.080*(0.302) |  |
| **KRD** |  |  | -0.435**(0.164) | -0.339*(0.167) | -0.294* (0.161) |
| **K10, score** |  |  |  | -0.078***(0.009) | -0.017***(0.002) |
| **Age group** | 60-74 | Reference | Reference | Reference | Reference |
|  | 75+ | -0.072 (0.121) | -0.075 (0.121) | -0.149 (0.124) | -0.027 (0.026) |
| **Gender** | Male | Reference | Reference | Reference | Reference |
|  | Female | -0.189 (0.108) | -0.191 (0.108) | -0.056 (0.111) | -0.018 (0.023) |
| **Educational attainment** | No school | Reference | Reference | Reference | Reference |
|  | Primary school | 0.172 (0.163) | 0.178 (0.163) | 0.158 (0.168) | 0.029 (0.034) |
|  | Junior school and above | 0.299 (0.174) | 0.310 (0.176) | 0.277 (0.180) | 0.045 (0.037) |
| **Marital status** | Married | Reference | Reference | Reference | Reference |
|  | Others | -0.050 (0.134) | -0.052 (0.134) | -0.135 (0.138) | -0.030 (0.029) |
| **Chronic disease** | No | Reference | Reference | Reference | Reference |
|  | Yes | -1.357***(0.112) | -1.355***(0.112) | -1.291*** (0.114) | -0.257*** (0.021) |
| **ADL group** | 14 | Reference | Reference | Reference | Reference |
|  | 15-21 | -0.762***(0.150) | -0.760***(0.150) | -0.638*** (0.155) | -0.133*** (0.033) |
|  | ≥22 | -1.932***(0.408) | -1.931***(0.409) | -1.710*** (0.413) | -0.317*** (0.061) |
| **Personality trait** | Introversion | Reference | Reference | Reference | Reference |
|  | Extraversion | 0.359**(0.113) | 0.358**(0.113) | 0.226* (0.117) | 0.049* (0.024) |
|  | In between | -0.058 (0.134) | -0.058 (0.134) | -0.173 (0.138) | -0.035 (0.029) |

KRD, Kakwani Relative Deprivation; K10, The Kessler Psychological Distress Scale

The coefficients in Model 1, 2, 3 were estimated by Binary logistic regression model and the coefficients in Model 4 was estimated by semiparametric regression model. Standard errors are in parentheses. *P <.05; **P <.01; ***P <.001.

Table B3 Association of absolute income and relative deprivation with SRH in rural older populations

| **Variable** |  | **Model 5** | **Model 6** | **Model 7** | **Model 8** |
| --- | --- | --- | --- | --- | --- |
| **Log income** |  | 0.203*(0.083) | -1.183***(0.185) | -1.131***(0.188) |  |
| **KRD** |  |  | -3.026***(0.347) | -2.736***(0.353) | -0.504***(0.062) |
| **K10, score** |  |  |  | -0.073***(0.005) | -0.013***(0.001) |
| **Age group** | 60-74 | Reference | Reference | Reference | Reference |
|  | 75+ | 0.234** (0.081) | 0.284** (0.082) | 0.179* (0.084) | 0.030 (0.016) |
| **Gender** | Male | Reference | Reference | Reference | Reference |
|  | Female | -0.028 (0.069) | 0.019 (0.070) | 0.094 (0.072) | 0.016 (0.014) |
| **Educational attainment** | No school | Reference | Reference | Reference | Reference |
|  | Primary school | 0.060 (0.073) | 0.027 (0.074) | 0.059 (0.075) | 0.013 (0.015) |
|  | Junior school and above | 0.090 (0.097) | 0.012 (0.098) | 0.047 (0.100) | 0.012 (0.020) |
| **Marital status** | Married | Reference | Reference | Reference | Reference |
|  | Others | -0.035 (0.082) | -0.057 (0.083) | -0.109 (0.085) | -0.021 (0.017) |
| **Chronic disease** | No | Reference | Reference | Reference | Reference |
|  | Yes | -1.478*** (0.072) | -1.412*** (0.073) | -1.326*** (0.074) | -0.274*** (0.014) |
| **ADL,score** | 14 | Reference | Reference | Reference | Reference |
|  | 15-21 | -1.095*** (0.081) | -1.063*** (0.082) | -0.958*** (0.083) | -0.197*** (0.016) |
|  | ≥22 | -1.992*** (0.183) | -1.904*** (0.185) | -1.619*** (0.190) | -0.264*** (0.025) |
| **Personality trait** | Introversion | Reference | Reference | Reference | Reference |
|  | Extraversion | 0.539*** (0.072) | 0.543*** (0.073) | 0.410*** (0.075) | 0.079*** (0.015) |
|  | In between | 0.326*** (0.090) | 0.319*** (0.091) | 0.218* (0.093) | 0.041* (0.018) |

KRD, Kakwani Relative Deprivation; K10, The Kessler Psychological Distress Scale

The coefficients in Model 5, 6, 7 were estimated by Binary logistic regression model and the coefficients in Model 8 was estimated by semiparametric regression model. Standard errors are in parentheses. *P <.05; **P <.01; ***P <.001.

Table B4 Sensitive analysis in urban older population

| **Variable** |  | **Model 2** | **Model 3** | **Model 4** |
| --- | --- | --- | --- | --- |
| **Log income** |  | -0.566*(0.225) | -0.276*(0.239) |  |
| **KRD** |  | -0.952**(0.396) | -0.874**(0.403) | -0.179**(0.161) |
| **K10, score** |  |  | -0.078***(0.009) | -0.017***(0.002) |
| **Age group** | 60-74 | Reference | Reference | Reference |
|  | 75+ | -0.077 (0.121) | -0.151 (0.124) | -0.025 (0.026) |
| **Gender** | Male | Reference | Reference | Reference |
|  | Female | -0.164 (0.109) | -0.032 (0.112) | -0.011 (0.023) |
| **Educational attainment** | No school | Reference | Reference | Reference |
|  | Primary school | -0.155 (0.163) | -0.139 (0.167) | 0.020 (0.035) |
|  | Junior school and above | -0.285 (0.173) | -0.256 (0.178) | 0.031 (0.037) |
| **Marital status** | Married | Reference | Reference | Reference |
|  | Others | -0.072 (0.134) | -0.152 (0.138) | -0.032 (0.029) |
| **Chronic disease** | No | Reference | Reference | Reference |
|  | Yes | -1.338*** (0.112) | -1.276*** (0.114) | -0.254*** (0.021) |
| **ADL group** | 14 | Reference | Reference | Reference |
|  | 15-21 | -0.746*** (0.151) | -0.625*** (0.155) | -0.132*** (0.033) |
|  | ≥22 | -1.960***(0.410) | -1.743*** (0.414) | -0.326*** (0.061) |
| **Personality trait** | Introversion | Reference | Reference | Reference |
|  | Extraversion | 0.348** (0.113) | 0.217* (0.117) | 0.046* (0.024) |
|  | In between | -0.053 (0.134) | -0.169 (0.138) | -0.033 (0.029) |

KRD, Kakwani Relative Deprivation; K10, The Kessler Psychological Distress Scale

The reference group was town/ sub-district. The coefficients in Model 2, 3 were estimated by logistic regression model and the coefficients in Model 4 was estimated by semiparametric regression model. Standard errors are in parentheses. *P <.05; **P <.01; ***P <.001.

Table B5 Sensitive analysis in rural older population

| **Variable** |  | **Model 6** | **Model 7** | **Model 8** |
| --- | --- | --- | --- | --- |
| **Log income** |  | -0.343**(0.133) | -0.315*(0.136) |  |
| **KRD** |  | -1.237***(0.232) | -0.992**(0.238) | -0.252***(0.046) |
| **K10, score** |  |  | -0.074***(0.005) | -0.014***(0.001) |
| **Age group** | 60-74 | Reference | Reference | Reference |
|  | 75+ | 0.282** (0.082) | 0.170* (0.084) | 0.030 (0.016) |
| **Gender** | Male | Reference | Reference | Reference |
|  | Female | 0.041 (0.071) | 0.107 (0.073) | 0.018 (0.014) |
| **Educational attainment** | No school | Reference | Reference | Reference |
|  | Primary school | 0.148* (0.075) | 0.160* (0.076) | 0.031* (0.015) |
|  | Junior school and above | 0.164 (0.098) | 0.177 (0.100) | 0.036 (0.020) |
| **Marital status** | Married | Reference | Reference | Reference |
|  | Others | -0.032 (0.082) | -0.090 (0.084) | -0.017 (0.017) |
| **Chronic disease** | No | Reference | Reference | Reference |
|  | Yes | -1.482*** (0.072) | -1.387*** (0.074) | -0.287*** (0.014) |
| **ADL,score** | 14 | Reference | Reference | Reference |
|  | 15-21 | -1.087*** (0.081) | -0.980 (0.083) | -0.204*** (0.016) |
|  | ≥22 | -1.927*** (0.184) | -1.638 (0.189) | -0.275*** (0.025) |
| **Personality trait** | Introversion | Reference | Reference | Reference |
|  | Extraversion | 0.534*** (0.073) | 0.401*** (0.075) | 0.079*** (0.015) |
|  | In between | 0.330*** (0.091) | 0.228* (0.093) | 0.043* (0.018) |

KRD, Kakwani Relative Deprivation; K10, The Kessler Psychological Distress Scale

The reference group was town sub-district. The coefficients in Model 6, 7 were estimated by logistic regression model and the coefficients in Model 8 was estimated by semiparametric regression model. Standard errors are in parentheses. *P <.05; **P <.01; ***P <.001.

Additional file 3: Regression results of logistic models and semiparametric model(1=very good/good/fair; 0=poor/very poor)

Table C1 Association of absolute income and relative deprivation with SRH in urban older populations

| **Variable** |  | **Model 1** | **Model 2** | **Model 3** | **Model 4** |
| --- | --- | --- | --- | --- | --- |
| **Log income** |  | 0.401** (0.138) | -0.706 (0.392) | -0.683 (0.407) |  |
| **KRD** |  |  | -1.823** (0.617) | -1.837** (0.637) | -0.389** (0.142) |
| **K10, score** |  |  |  | -0.089*** (0.011) | -0.012*** (0.002) |
| **Age group** | 60-74 | Reference | Reference | Reference | Reference |
|  | 75+ | -0.075 (0.176) | -0.109 (0.176) | -0.242 (0.180) | -0.018 (0.018) |
| **Gender** | Male | Reference | Reference | Reference | Reference |
|  | Female | -0.123 (0.164) | -0.145 (0.165) | 0.050 (0.171) | 0.006 (0.015) |
| **Educational attainment** | No school | Reference | Reference | Reference | Reference |
|  | Primary school | 0.010 (0.229) | -0.029 (0.230) | -0.011 (0.236) | 0.006 (0.025 |
|  | Junior school and above | -0.225 (0.248) | -0.302 (0.251) | -0.255 (0.258) | -0.009 (0.026) |
| **Marital status** | Married | Reference | Reference | Reference | Reference |
|  | Others | -0.034 (0.195) | -0.075 (0.196) | -0.199 (0.203) | -0.019 (0.020) |
| **Chronic disease** | No | Reference | Reference | Reference | Reference |
|  | Yes | -1.799*** (0.246) | -1.784*** (0.246) | -1.673*** (0.249) | -0.099*** (0.011) |
| **ADL group** | 14 | Reference | Reference | Reference | Reference |
|  | 15-21 | -0.803*** (0.188) | -0.792*** (0.188 | -0.623** (0.195) | -0.074** (0.027) |
|  | ≥22 | -1.953*** (0.326) | -1.960*** (0.327) | -1.727*** (0.341) | -0.315*** (0.070) |
| **Personality trait** | Introversion | Reference | Reference | Reference | Reference |
|  | Extraversion | 0.339* (0.161) | 0.336* (0.162) | 0.156 (0.167) | 0.017 (0.017) |
|  | In between | 0.469* (0.201) | 0.461* (0.201) | 0.340 (0.208) | 0.033 (0.019) |

KRD, Kakwani Relative Deprivation; K10, The Kessler Psychological Distress Scale

The coefficients in Model 1, 2, 3 were estimated by Binary logistic regression model and the coefficients in Model 4 was estimated by semiparametric regression model. Standard errors are in parentheses. *P <.05; **P <.01; ***P <.001.

Table C2 Association of absolute income and relative deprivation with SRH in rural older populations

| **Variable** |  | **Model 5** | **Model 6** | **Model 7** | **Model 8** |
| --- | --- | --- | --- | --- | --- |
| **Log income** |  | 0.624*** (0.111) | -1.434*** (0.164) | -1.421*** (0.167) |  |
| **KRD** |  |  | -3.358*** (0.324) | -3.092*** (0.330) | -0.565*** (0.064) |
| **K10, score** |  |  |  | -0.068*** (0.005) | -0.012*** (0.001) |
| **Age group** | 60-74 | Reference | Reference | Reference | Reference |
|  | 75+ | 0.289** (0.096) | 0.298** (0.097) | 0.171 (0.099) | 0.030* (0.014) |
| **Gender** | Male | Reference | Reference | Reference | Reference |
|  | Female | 0.086 (0.084) | 0.079 (0.085) | 0.159 (0.087) | 0.022 (0.012) |
| **Educational attainment** | No school | Reference | Reference | Reference | Reference |
|  | Primary school | -0.029 (0.086) | -0.040 (0.087) | 0.001 (0.089) | -0.001 (0.012) |
|  | Junior school and above | 0.147 (0.121) | 0.152 (0.122) | 0.232 (0.126) | 0.022 (0.016) |
| **Marital status** | Married | Reference | Reference | Reference | Reference |
|  | Others | -0.036 (0.097) | -0.046 (0.098) | -0.114 (0.100) | -0.016 (0.014) |
| **Chronic disease** | No | Reference | Reference | Reference | Reference |
|  | Yes | -1.445*** (0.110) | -1.364*** (0.111) | -1.252*** (0.113) | -0.125*** (0.010) |
| **ADL,score** | 14 | Reference | Reference | Reference | Reference |
|  | 15-21 | -0.974*** (0.085) | -0.945*** (0.086) | -0.820*** (0.089) | -0.132*** (0.016) |
|  | ≥22 | -1.891*** (0.142) | -1.833*** (0.144) | -1.509*** (0.150) | -0.289*** (0.030) |
| **Personality trait** | Introversion | Reference | Reference | Reference | Reference |
|  | Extraversion | 0.423*** (0.084) | 0.434*** (0.085) | 0.273** (0.088) | 0.039** (0.013) |
|  | In between | 0.566*** (0.108) | 0.555*** (0.110) | 0.423*** (0.112) | 0.064*** (0.015) |

KRD, Kakwani Relative Deprivation; K10, The Kessler Psychological Distress Scale

The coefficients in Model 5, 6, 7 were estimated by Binary logistic regression model and the coefficients in Model 8 was estimated by semiparametric regression model. Standard errors are in parentheses. *P <.05; **P <.01; ***P <.001.
